# Clinical and Molecular Epidemiology of Crimean-Congo Hemorrhagic Fever in Humans in Uganda, 2013–2019

**DOI:** 10.4269/ajtmh.21-0685

**Published:** 2021-10-18

**Authors:** Stephen Balinandi, Shannon Whitmer, Sophia Mulei, Luke Nyakarahuka, Alex Tumusiime, Jackson Kyondo, Jimmy Baluku, Joseph Mutyaba, Lawrence Mugisha, Maja Malmberg, Julius Lutwama, Trevor R. Shoemaker, John D. Klena

**Affiliations:** ^1^ Department of Arbovirology, Emerging and Re-emerging Infectious Diseases, Uganda Virus Research Institute, Entebbe, Uganda;; ^2^ Viral Special Pathogens Branch, Centers for Disease Control and Prevention, Atlanta, Georgia, USA;; ^3^ School of Veterinary Medicine and Animal Resources, College of Veterinary Medicine, Animal Resources and Biosecurity, Makerere University, Kampala, Uganda;; ^4^ EcoHealth Research Group, Conservation & Ecosystem Health Alliance (CEHA), Kampala, Uganda;; ^5^ Section of Virology, Department of Biomedical Sciences and Veterinary Public Health, Swedish University of Agricultural Sciences, Uppsala, Sweden;; ^6^ SLU Global Bioinformatics Centre, Department of Animal Breeding and Genetics, Swedish University of Agricultural Sciences, Uppsala, Sweden;; ^7^ School of Biosecurity, Biotechnical and Laboratory Sciences, College of Veterinary Medicine, Animal Resources and Biosecurity, Makerere University, Kampala, Uganda

## Abstract

Crimean-Congo Hemorrhagic Fever (CCHF) is endemic in Uganda, yet its epidemiology remains largely uncharacterized. To better understand its occurrence within Uganda, case reports of patients hospitalized with CCHF between 2013 and 2019 were reviewed. Further, genome sequences of CCHF-positive RNA obtained during this period were determined for phylogenetic comparisons. We found that a total of 32 cases (75% males; CFR, 31.2%), aged between 9 to 68 years, were reported during the study period. Most cases were detected during July to December of each outbreak year (81.2%; *P* < 0.01) and were located along the “cattle corridor” (68.7%, *P* = 0.03). The most common presenting symptoms were fever (93.8%), hemorrhage (81.3%), headache (78.1%), fatigue (68.8%), vomiting (68.8%), and myalgia (65.6%). In five patients for whom hematological data were available, varied abnormalities were observed including thrombocytopenia, leukopenia, anemia, lymphopenia, lymphocytosis, polycythemia, and microcytosis. About 56.3% (*P* = 0.47) of patients reported tick bites or exposure to livestock as their potential source of infection. Person-to-person transmission was suspected for two cases. Using unbiased metagenomics, we found that the viral S- and L- segments have remained conserved in Africa 2 clade since the 1950s. In contrast, the M segment split into two geographically interspersed clades; one that belongs to Africa 2 and another that is ancestral to Africa 1 and 2. Overall, this data summarizes information on the history and clinical presentation of human CCHF in Uganda. Importantly, it identifies vulnerable populations as well as temporal and geographic regions in Uganda where surveillance and control interventions could be focused.

## INTRODUCTION

Crimean-Congo Hemorrhagic Fever (CCHF) is a tick-borne disease, broadly distributed in Eastern Europe, Asia, Middle East, and Africa where it affects both animals and humans.^1^ Among humans, it was first clinically described during the 1940s when Russian troops reoccupying the Crimean Peninsula presented with a hemorrhagic disease.^2^ In the 1950s, an identical virus was found to be the cause of illness among patients in the north-eastern parts of the present-day Democratic Republic of Congo—hence the adoption of its current name “Crimean-Congo Hemorrhagic Fever.”^3^ Although CCHF infection in animals is asymptomatic, it can manifest as a sudden onset of fever, headache, muscle pain, vomiting, and hemorrhage in about one in five of infected humans,^4^^,^^5^ with death occurring in about 15% or more of hospitalized individuals.^6^^,^^7^ Additionally, with the international concern that CCHF attracts, a single human case in Uganda constitutes an outbreak, and is mandatorily reported to WHO under the International Health Regulations as amended in 2005.^8^^,^^9^

The etiological agent of CCHF is an enveloped, negative-sense RNA Orthonairovirus of the Nairoviridae family designated Crimean-Congo Hemorrhagic Fever Virus (CCHFV).^10^ Although CCHFV strains have been detected in many species of hard ticks, the main reservoir and vector are *Hyalomma* spp. ticks.^3^ Humans become infected through tick bites, but infection can also occur after close contact with infectious materials.^11^ The most-at-risk persons for CCHF infection include individuals working in agricultural fields, abattoirs, and herdsmen who may be exposed to ticks as well as infectious animal products such as milk and meat. Secondary transmissions are also common among medical staff, other hospital patients and family members and relatives who become exposed through infectious blood or body fluids of acutely sick patients.^12^

Crimean-Congo Hemorrhagic Fever is well-studied in Europe and Asia,^13^ however its epidemiology in sub-Saharan Africa is much less defined,^14^ with most available country information having been derived from human and animal serological studies and detection in *Hyalomma* spp. ticks.^15^^–^^17^ Because of its potential for use as an agent of bioterror^18^ and the lack of approved therapeutics or vaccines, CCHF is listed by WHO as a priority emerging disease requiring accelerated efforts in surveillance, research, and diagnostics development.^19^

In Uganda, CCHF surveillance was initiated in May 2010, as part of the efforts to strengthen the national capacity to control viral hemorrhagic fevers. In 2013, the surveillance system was enhanced through efforts to improve infectious and zoonotic disease outbreak detection and response.^20^^,^^21^ As a result, after almost 20 years of no human CCHF case reports, the first cases of CCHF were detected in 2013.^21^ This new and enhanced surveillance activity is part of the National Viral Hemorrhagic Fever (VHF) surveillance system that also tests for filoviruses (e.g., Ebola spp. and Marburg) and Rift Valley Fever virus (RVF).^22^^,^^23^ To further understand the current epidemiological situation of CCHF in Uganda, we have reviewed the CCHF surveillance and outbreak investigation data collected between 2013 and 2019, and summarize the clinical, laboratory, and epidemiological aspects observed in hospitalized patients during this period. We also provide whole genome sequence information characterizing strains of CCHFV resulting in human disease in Uganda. Analysis of the surveillance, laboratory, and genetic data will be useful for creating early warning systems for CCHF and other VHF diseases in Uganda; this information can also be used in developing health education messages and preventive intervention strategies.

## MATERIALS AND METHODS

### Ethical statement.

As all the reviewed data were collected as part of routine healthcare and mandated surveillance, or as part of confirmed outbreak investigations, the Ugandan Ministry of Health guidelines did not require ethical review, or personal consent, as these activities are considered events of public health concern that require immediate response and containment.^24^

### Surveillance process and data collection.

The operational structure of the Ugandan VHF surveillance and laboratory program has been previously described.^21^ Briefly, samples from suspected VHF cases presenting in health facilities across the country, are collected by trained healthcare workers and appropriately transported mainly through a courier network,^25^ to Uganda Virus Research Institute (UVRI), Entebbe, Uganda, where a high-containment, BSL-3 diagnostics laboratory has been established since 2010. This laboratory uses both serological and molecular techniques for CCHF and other VHF diagnostics.^21^^,^^26^ The working surveillance case definition for suspected VHF cases is any individual presenting with acute onset of fever (≥ 38.0°C), with no alternative diagnosis (e.g., malaria, typhoid), and presenting with any four of the following signs and symptoms: intense fatigue, chills, abdominal pains, headache, arthralgia, myalgia, anorexia, vomiting, diarrhea, skin rash, jaundice, and unexplained bleeding from any site.

When samples are collected from suspected patients (preferred samples are whole blood, plasma, or serum), a standardized case report form (CRF) is also completed and transported to the laboratory along with the samples. All suspected VHF samples are tested by reverse transcription polymerase chain reaction (RT-PCR) for Ebola viruses, Marburg virus, CCHF virus, and RVF virus to confirm acute infection, using protocols as previously reported.^26^ Samples may also be examined using virus-specific IgM ELISA assays developed by the US CDC. If acute VHF infection is confirmed, field investigation and clinical case management teams are deployed to the outbreak area and are mandated to collect additional epidemiological and clinical information about the index, and other case patients, and document potential exposure(s) that led to infection.^24^

### CCHFV qRT-PCR, RNA sequencing, and bioinformatics.

All laboratory procedures performed at UVRI for the confirmation of CCHFV infection in suspected patients have been previously described.^26^ Briefly, all samples in this study were tested for CCHFV using RT-PCR and in some cases supported by using an ELISA detecting ant-CCHF IgM antibodies. This combination of diagnostic approaches is widely used in CCHF-related studies.^27^ Specimens that were RT-PCR positive for CCHFV with Ct values < 32, were prepared for sequencing at UVRI or the United States Centers for Disease Control and Prevention (CDC, Atlanta, GA) using unbiased or CCHFV-enrichment techniques.^28^^,^^29^ Whole blood specimens were inactivated with Tripure (Roche, Basel, Switzerland) or 5X Magmax™ 96 Viral Isolation kit (Applied Biosystems Inc., Vilnius, Lithuania).^30^^,^^31^ Ribonucleic acid was extracted by phase-separation using 1-bromo-3-chloropropane (Sigma-Aldrich, St. Louis, MO) and applied to Clean and Concentrate-25 columns (Zymo Research, Irvine, CA) for further purification and concentration. Extracted RNA was treated with RNase-free DNase (Roche, Basel, Switzerland) and prepared for unbiased next generation sequencing (NGS) using a TruSeq RNA Access Library preparation kit (Illumina, San Diego, CA) with CCHFV-specific enrichment oligos. Next generation sequencing libraries were also prepared using NEBNext Ultra II Directional RNA library preparation kit (New England Biolabs, Beverly, MA). Libraries were sequenced using either an Illumina iSeq100 (V1 2 × 150 cycles), MiSeq or MiniSeq (High Output 2 × 150 cycles).^32^ An initial complete Uganda CCHFV genome was generated using viral-ngs (Broad Institute, Boston, MA; https://viral-ngs.readthedocs.io/en/latest/) with a custom CCHFV-lastal database. Reads from subsequent specimens were mapped to the complete Uganda CCHFV genome using in-house scripts-consisting of quality trimming (printseq-lite -min_qual_mean 25 -trim_qual_right 20 -min_len 50), read mapping (BWA-mem),^33^ and PCR-de-duplication (picard MarkDuplicates; http://picard.sourceforge.net). Consensus genomes were called using Geneious software (threshold = 0%, Assign Quality = total, minimum coverage > 2; v10).^34^ Crimean-Congo Hemorrhagic Fever virus genomes were also *de novo* assembled using SPAdes (-k auto, v3.14.0).^35^ To improve genome coverage, partial genomes and contigs were blasted to identify more closely related reference sequences. All genomes were iteratively assembled three times to incorporate any minor variants relative to the initial reference (or *de novo* contig) sequence. Evolutionary history was inferred using all available full-length CCHFV genomes from GenBank using raxml (-m GTRGAMMA -p $RANDOM -f a -x $RANDOM -N 1000) with bootstrap support provided by 1,000 iterations.^36^ Evidence of recombination was assessed with Recombination Detection Program (v5.05)^37^ using RDP, GENECONV, BootScan, MaxChi, Chimaera, SiScan, and 3Seq with alignments of Uganda CCHF genomes. Crimean-Congo Hemorrhagic Fever Virus genomes were deposited to GenBank and given accession numbers as follows: MW464961, MW464985, MW464976, MW464952, MW464958, MW464967, MW464979, MW464949, MW464964, MW464946, MW464969, MW464955, MW464982, MW464972 (Figure 1A); MW464959, MW464953, MW464977, MW464980, MW464950, MW464962, MW464965, MW464947, MW464970, MW464956, MW464983, MW464973 (Figure 1B) and MW464945, MW464944, MW464948, MW464971, MW464966, MW464974, MW464984, MW464957, MW464975, MW464963, MW464986, MW464951, MW464943, MW464968, MW464981, MW464978, MW464960, MW464954 (Figure 1C).

**Figure 1.**
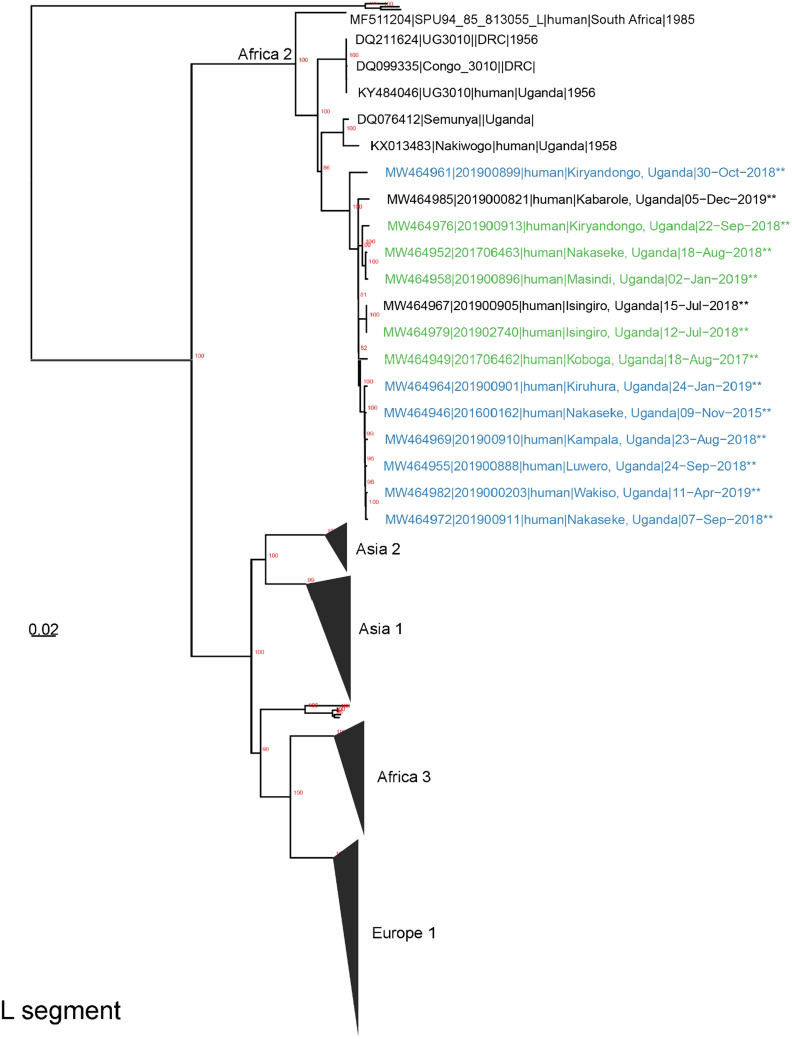
Inferred phylogenetic history of all nearly full-length Crimean-Congo Hemorrhagic Fever (CCHF) Orthonairovirus species using maximum likelihood estimation. Bootstrap support (*N* = 1,000 iterations) greater than 70% is labeled red and trees are midpoint rooted. Major geographic clades are labeled, and new genomes are indicated by ** at the end of sequence names. (**A**) The CCHF L segments from Uganda are monophyletic. L segment tree consists of *N* = 198 total and 14 new genomes. Sequence names are colored according to M segment clades. (**B**) The CCHF M segments from Uganda form two distinct monophyletic clades (highlighted green and blue). M segment tree consists of *N* = 218 total and 12 new genomes. (**C**) The S segments form two monophyletic clades consisting of older (2013) and more recent sequences (2013–2019). Tree contains *N* = 259 total and 18 new genomes. Sequence names are colored according to M segment clades. This figure appears in color at www.ajtmh.org.

**Figure 1.**
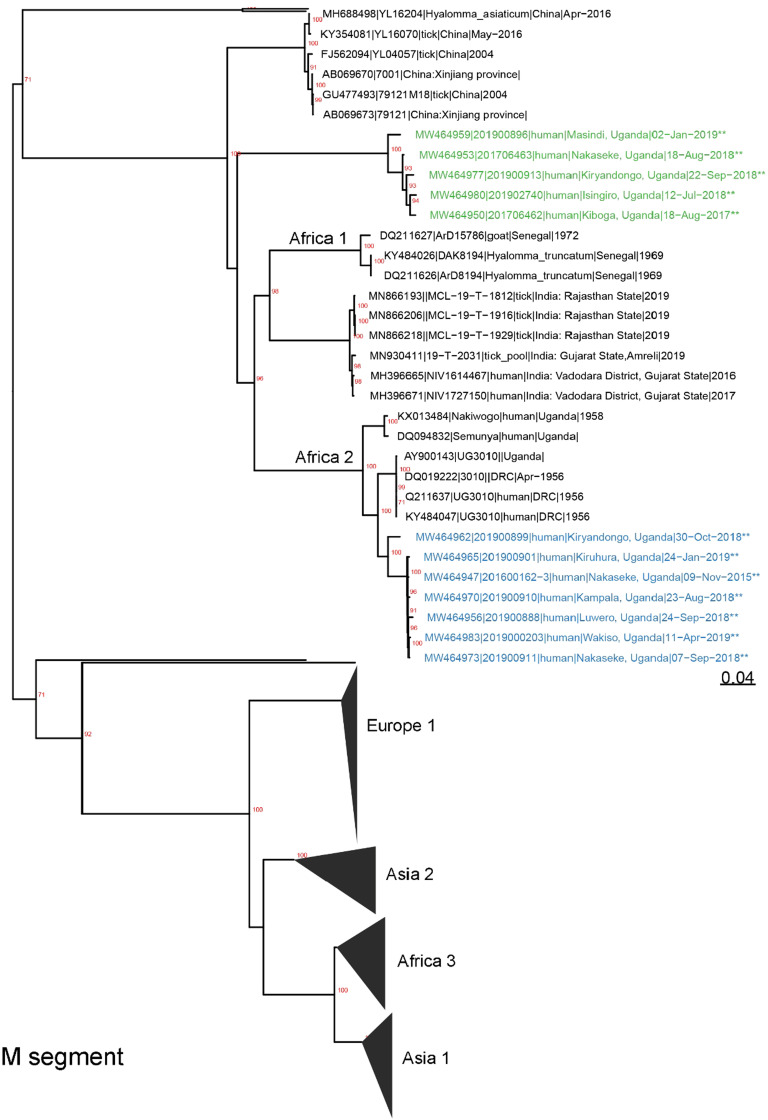
Continued.

**Figure 1.**
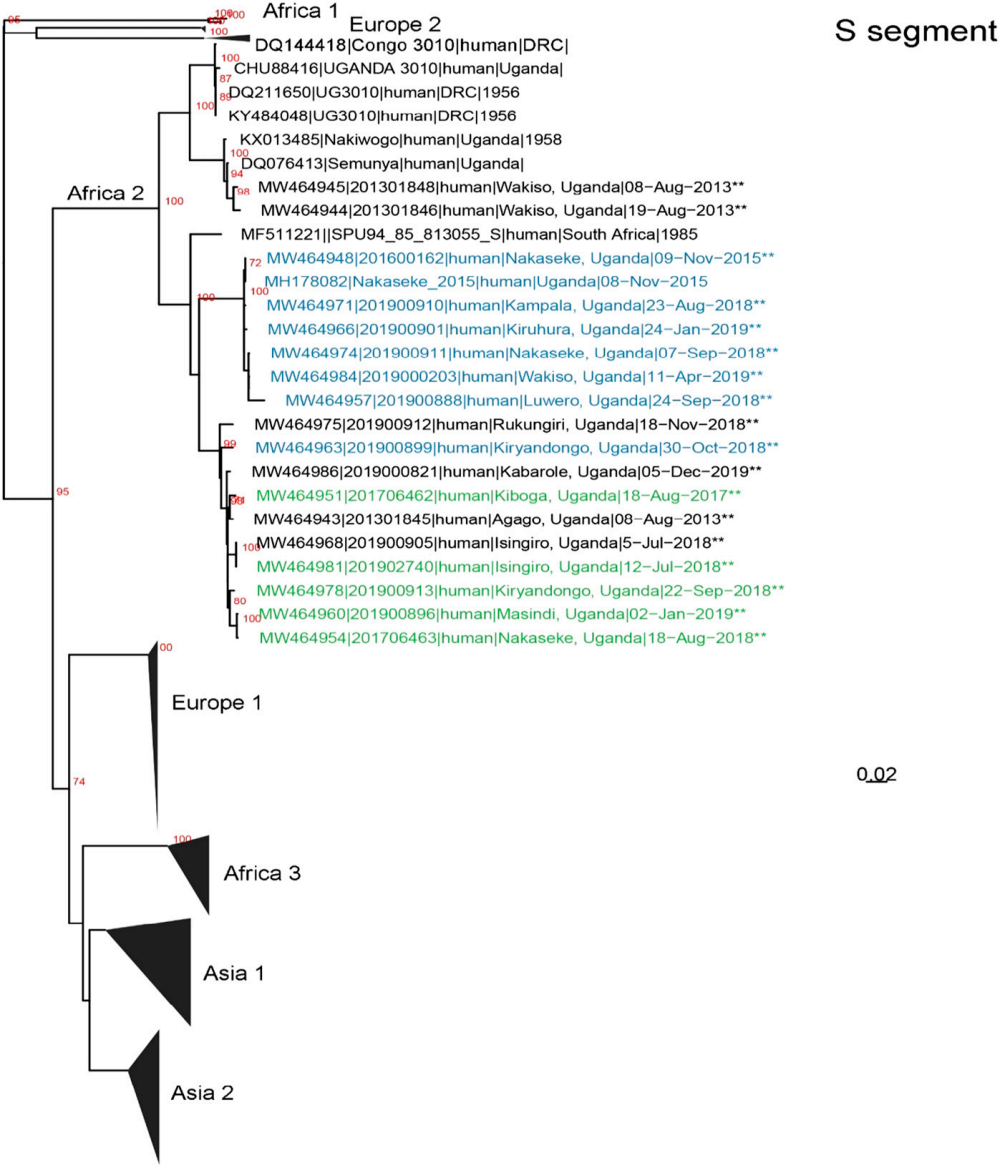
Continued.

### Statistical analysis.

Case variables that were considered for this study included demographic (age, sex, and occupation), clinical (date of disease onset, presenting signs and symptoms, hematologic profiles), geographical location (district, agroecological zone), exposure and illness outcome data. Data was abstracted from CRFs and summarized into MS Excel (Microsoft Corporation, Redmond, WA). Thereafter, categorical variables were descriptively analyzed in form of frequency and percent distributions, while means and/or medians were computed for continuous data. Statistical comparisons were performed using MedCalc software,^38^ with a *P*-value < 0.05 being considered statistically significant.

## RESULTS

### Summary of outbreaks and patient demographics.

**Figure 2.**
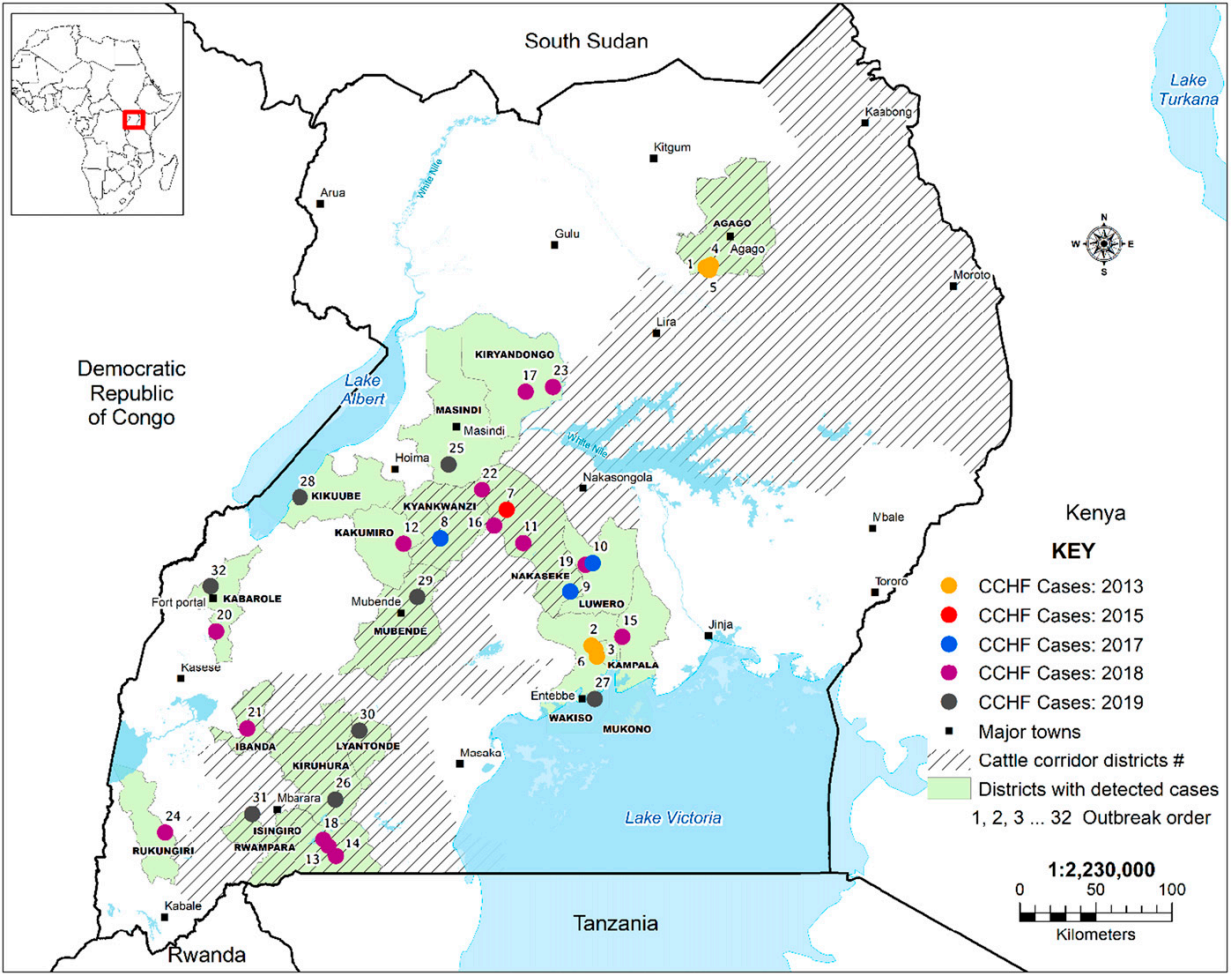
Location of Crimean-Congo Hemorrhagic Fever (CCHF) cases, Uganda, 2013–2019; #Cattle Corridor map was adapted from Sempiira et al.,^41^ with slight modification. This figure appears in color at www.ajtmh.org.

From May 2013 to December 2019, the UVRI VHF laboratory received samples from a total of 1,261suspected VHF cases, out of which, 28 independent CCHF outbreaks were detected involving 32 confirmed cases (Figure 2). Of these cases, 31.2% (*N* = 10; six males and four females) died during their CCHF illness. The first two outbreaks were confirmed concurrently in Agago (three cases) and Wakiso (two cases) districts during August 2013. These districts are separated by a distance of greater than 400 km, and no known epidemiological linkage between the cases in these two outbreaks could be established. A third independent outbreak of CCHF was detected on December 17, 2013; CCHFV was detected in a sample obtained from a 50-year-old woman admitted to Case Medical Center, a private health clinic within Kampala city. The next outbreak, detected on November 9, 2015, involved a 33-year-old male para-veterinarian from Nakaseke district.^26^ No additional CCHF cases were detected for another 2 years until August 2017, when there were two additional independent outbreaks occurring concurrently in the neighboring districts of Kyankwanzi and Nakaseke.^39^ The two cases had no epidemiological linkage. A few months later, on December 26, 2017, a 9-year-old boy from Luweero district was admitted to Kiwoko hospital and confirmed as the 10th CCHF case. During the subsequent two years, 2018 to 2019, 18 additional outbreaks involving 22 confirmed CCHF cases were detected,^40^ with the last CCHF case in 2019 being a 25-year-old female from Kabarole district, who was identified retrospectively at Fort-Portal Regional Referral Hospital on December 5, 2019.

As shown in Figure 2, CCHF confirmed cases in Uganda were detected from various districts, 68.7% (*N* = 22) of which are located within the “Cattle Corridor” zone (*P* = 0.03). In fact, except for one case who lived in Kampala City, the majority of cases occurred in rural locations, in persons practicing subsistent crop farming and/or livestock keeping (50.0%), students (18.8%), and individuals engaged in local business ventures (12.5%) (Table 1). About 56.3% (*N* = 18; *P* = 0.47) of cases reported that they were exposed to ticks or livestock animals in their routine livelihoods. Only two cases (both female), from Wakiso and Isingiro districts, appeared to have been probable secondary transmissions given that their onset of disease was within a few days after they extensively interacted with an index family member.

**Table 1:** Demographical and clinical characteristics of hospitalized CCHF patients, Uganda, 2013–2019

Characteristic		Frequency	%
Gender	Male	24	75.0
	Female	8	25.0
	Farmer or herdsperson	16	50.0
	Child or student	6	18.8
Occupation	Business	4	12.5
	Housewife	2	6.3
	Refugee	2	6.3
	Teacher	1	3.1
	Unknown	1	3.1
Age in years	Range; Mean	6–68; 28	
Age in distribution in years	0–10	4	12.5
	11–20	6	18.8
	21–30	10	31.3
	31–40	7	21.9
	41–50	4	12.5
	> 51	1	3.1
Clinical outcome	Alive	22	68.8
	Died	10	31.2
Main clinical signs and symptoms	Fever	30	93.8
	Hemorrhage	26	81.3
	Headache	25	78.1
	Fatigue	22	68.8
	Vomiting	22	68.8
	Myalgia	21	65.6
	Anorexia	17	53.1
	Stomachache	17	53.1
	Diarrhea	16	50.0
	Coughing	9	28.1
	Conjunctivitis	6	18.8
	Delirium	5	15.6
	Sore throat	4	12.5
	Dyspnea	4	12.5
	Skin rash	3	9.4
	Dysphagia	1	3.1
Potential exposure to livestock and ticks	Yes	18	56.3
	No	12	37.5
	Unknown	2	6.3
Residence located within “Cattle Corridor” zone	Yes	22	68.7
	No	10	31.3

**Figure 3.**
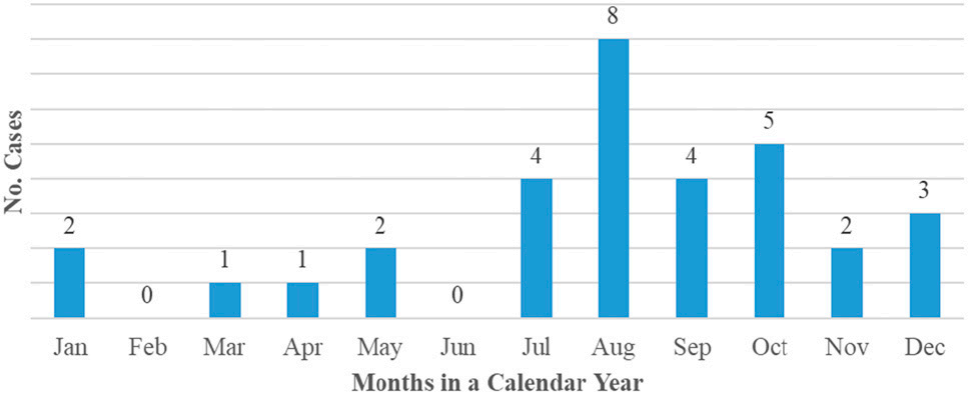
Cumulative incidence of Crimean-Congo Hemorrhagic Fever (CCHF) cases detected during each month in a calendar year, Uganda, 2013–2019. This figure appears in color at www.ajtmh.org.

All laboratory-confirmed CCHF cases were aged between 9 and 68 years (Mean: 28; Median: 30), with 65.5% (*N* = 21) aged between 21 and 50 years. Seventy-five percent of the cases were male (*N* = 24; *P* < 0.01). Crimean-Congo Hemorrhagic Fever cases in Uganda have been recorded in almost every month in a calendar year, with a rise in cases starting around July, peaking during August, and lowering toward the end of the year (Figure 3). Over the study period, 81.2% of outbreaks occurred from July to December (*P* < 0.01).

### Clinical and hematological characteristics of cases.

Fever was the most common presenting clinical symptom (93.8%), followed by bleeding or hemorrhage (81.3%), headache (78.1%), fatigue (68.8%), vomiting (68.8%), and myalgia (65.6%). Skin rash (9.4%) and difficulty in swallowing (3.1%) were the least reported clinical symptoms among CCHF cases in Uganda during the study period.

Field hematological investigations in CCHF cases are not recommended due to the associated health risks to laboratory workers.^42^ However, due to the atypical nature of the disease especially during the early phase(s) of illness, clinicians may inadvertently include these investigations.^43^ In our study, we found hematological data for five cases, which had been obtained at the time of their hospital admission before a VHF diagnosis was made. As shown in Table 2, case hematological profiles had varied abnormalities including leukopenia (*N* = 3), anemia (2), lymphopenia (*N* = 2), polycythemia (*N* = 1), lymphocytosis (*N* = 1), and microcytosis (*N* = 1). All cases presented with thrombocytopenia (100%).

**Table 2:** Parameters associated with hematological abnormalities among hospitalized CCHF patients, Uganda, 2013–2019

Parameter	Median [mean]	Observed range	Normal range*
White blood cells [10^3^ cells/µL]	6.1 [6.1]	1.2–11	2.8–8.2
Lymphocyte cells [10^3^ cells/µL]	1.3 [1.9]	0.7–4.4	1.2–3.7
Monocyte cells [10^3^ cells/µL]	0.5 [0.7]	0.11–1.6	0.2–0.7
Monocyte [%]	9.2 [13.7]	6.4–25.6	4.7–12.7
Granulocyte cells [10^3^ cells/µL]	2.9 [2.5]	0.42–4.1	2.5–7.5
Granulocyte [%]	44.8 [42.6]	35.0–47.9	50–75
Red blood cells [10^6^ cells/µL]	4.6 [4.6]	2.6–6.4	3.5–6.1
Hematocrit [%]	34.2 [33.3]	19.5–45.4	31.2–49.5
Hemoglobin [g/dL]	12.1 [12.4]	6.5–17.0	10.8–17.1
Platelets [10^3^ cells/µL]†	25.0 [32.4]	21–50	109–384

*Reference ranges previously determined for Ugandan adult blood donors.^44^

†All cases were below the normal range.

### Phylogenetic analysis.

In the current study, we attempted viral genetic sequencing on samples collected from a total of 20 cases, particularly those cases which were confirmed from 2017 to 2019. This represents 80% of all CCHF cases detected during that period of this study. As shown in Figure 1, the obtained sequences represent the L segment (*N* = 14), M segment (*N* = 12), and S segment (*N* = 18). One extra sequence, Accession number: MH178082 in Figure 1C, was a partial sequence obtained in 2015 using Sanger sequencing approach,^26^ but was repeated for whole genome sequencing under the present study and is shown with Accession Number: MW464948 in Figure 1C.

We observed that since 2013, CCHF in Uganda is closely related to the Africa 2 clade—either as a member of the clade (L, S, and M segments), or as an ancestral clade (M segment) (Figure 1). All L segments clustered together into the Africa 2 clade—a clade that also contains sequences collected from Uganda in 1956–1958. The S segments also contained members of the Africa 2 clade and in agreement with the L segment shared a most recent common ancestor with specimens collected in Uganda and South Africa during the 1950s and 1980s, respectively. Despite an apparent split in the S segment clade, this bifurcation was not strongly supported as the bootstrap value was less than 70%.^45^ In contrast, circulating CCHF strains were divided into two separate clades based on M segment analysis; one clade belonging to the Africa 2 lineage and another that is ancestral to the Africa 1 and 2 lineages. The separation of the M-specific clades was not correspondingly maintained for the L and S segments. Recombination analysis identified a potential recombinant, between the two Ugandan M clades (Supplemental Table 1)

## DISCUSSION AND CONCLUSION

Outbreaks of CCHF are often high profile and generate considerable international attention due to their potential to become public health events of international concern. In fact, CCHF outbreaks must be reported to WHO under the International Health Regulations (2005). In Uganda, cases of CCHF were detected in 5 of the 7 years from 2013 to 2019 (except for 2014 and 2016), with an increasing trend in annual cases observed after 2017. The increase in human CCHF cases over the last few decades is noted in many countries.^1^^,^^46^^,^^47^ So far, several studies have attributed the increase or emergence of CCHF cases in localities to climatic changes and other anthropogenic changes that alter the biology and environmental dynamics of the tick vector.^48^^,^^49^ In other countries, however, a higher number of observed CCHF cases has been attributed to improved surveillance and prompt diagnosis of suspected cases.^50^ Whereas it could be true for Uganda that CCHF is actually reemerging in the country, we also note that, in the recent past, there has been an increase in public awareness, improved surveillance, and training of healthcare workers about VHFs in the country.^21^^,^^51^^,^^52^ Specifically, Uganda’s disease surveillance activities during 2018 and 2019 were heightened through active surveillance and expedited sample transport due to an ongoing Ebola outbreak in the neighboring Democratic Republic of the Congo (DRC).^53^

As observed in similar studies,^6^^,^^54^ confirmed cases of CCHF in Uganda during the study period were disproportionately male (24 males versus eight females; *P* < 0.01). In previous studies,^55^^,^^56^ the risk for CCHFV infection increased among people working in butcheries and those who come in close contact with animals. As observed in many African pastoral traditions, the predisposition of men to CCHFV infection in Uganda is likely due to the patriarchal nature of animal husbandry practices in the country. This is further supported by the fact that a higher percentage (84.4%) of cases in our study were aged 21–50 years, which is traditionally the age group that performs most outdoor-related pastoral or animal husbandry activities. Meanwhile, the case fatality rate (CFR) in this study was 31.2%, which fell within the reported CFR range of 5–50% among hospitalized cases in similar studies.^6^^,^^54^^,^^57^

As observed in this study, and elsewhere,^6^^,^^43^^,^^54^^,^^57^^,^^58^ clinical presentations between CCHF patients varied broadly from nonspecific manifestations, to severe multi-organ failure and fulminant hemorrhage. In our study, fever, hemorrhage, fatigue, muscle pains, vomiting, and anorexia were frequently observed in the patients. In addition, although the data is limited, thrombocytopenia was present in all cases. This manifestation is a common hemopathological abnormality in CCHF patients and has been reported elsewhere.^57^^,^^59^^,^^60^

Crimean-Congo Hemorrhagic Fever cases reported in this study showed both spatial and temporal relationships. A higher number of cases were not only located within the cattle corridor zone but were also detected during the months ending a calendar year (July to December). According to Wikel,^61^ the epidemiology and ecology of tick-borne agents in a locality is influenced by several factors such as weather and climatic conditions, anthropogenic changes, wildlife animal reservoirs, and some intrinsic changes within ticks and tick-borne agents. Indeed, most of these factors are not a rarity in the Ugandan cattle corridor—a large savannah grassland area that stretches diagonally from the North-Eastern to the South-Western parts of the country. This area is characterized by large swathes of low vegetation, long periods of uneven rainfall, and fragmented settlements. It is typically dry during the months of July and August of every year.^62^ Similar environmental correlates for a high number of CCHF cases have been found in Bulgaria,^49^ Iran,^63^ and Senegal.^64^

Phylogenetically, CCHFV circulating in Uganda since 2013 are closely related to the Africa 2 clade—either as a member of the clade (L, S, and M segments), or as an ancestral clade (M segment). Interestingly, the two M segment clades are geographically interspersed and, in some cases, circulate within the same districts (Kiryandongo, Nakaseke) or in neighboring districts (Kiruhura and Isingiro), demonstrating that a lack of geographic separation currently defines clade identity. Further supporting the idea that these two M clades are geographically intermingled, we observed a potential recombinant M segment between these two clades; however, there was low genome coverage over the proposed recombinant region. It is likely that the M segment clades may have originally been geographically separated, but external introductions, animal movement along the cattle corridor, or animal movement around the country, may have led to recent intermingling of these clades. Alternatively, these diverse M segments may reflect a carriage by different tick hosts as CCHFV has previously been detected from different tick species within Uganda.^3^^,^^26^^,^^65^

The Africa 2 clade contains sequences from specimens collected in Uganda and DRC from the 1950s and a single South African sequence collected in 1985.^65^ Thus, combined with data from this study, CCHFV has likely circulated undetected in Uganda in the human, animal, or arthropod populations from the 1950s, or earlier, and recent human cases have only been detected due to the enhanced activity of the National VHF surveillance system. This argument appears to be supported by data from our recent study in which CCHFV was determined to be widespread in Uganda, including in areas with no history of human cases.^17^ Furthermore, despite limited CCHFV sequences from neighboring countries, the close relationships of the Ugandan CCHFV sequences demonstrate that novel CCHFV variants, as far as we know, are unlikely being introduced into the country. This is in contrast to CCHFV sequences from Iran and Pakistan, where animal trade and movement is hypothesized to have led to the introduction of novel CCHFV variants.^66^^,^^67^

Overall, these data are suggestive of the most-at-risk persons, time periods of the year, specific geographic regions, and characteristics of the circulating virus which should be used for enhanced CCHF surveillance and control interventions in Uganda. We observe that most CCHF cases in Uganda have had direct contact or exposure to livestock animals and are mainly from the central region of Uganda. Given the sporadic and discreet nature of the outbreaks detected in Uganda during the study period, most of which were single case outbreaks, we hypothesize that tick-to-human transmission may be a common route of infection. In a previous study, we found that a high diversity of ticks was parasitizing domestic animals in Uganda.^68^ Therefore, application of routine and effective control measures that reduce the overall tick abundance in the environment is likely to have a significant reduction on the overall risk of CCHFV transmission to humans. Despite the limitation that the hematological data analyzed under this study refers to a small proportion of the studied cases, the presence of thrombocytopenia as a common clinical manifestation among cases could be a useful indication for increased suspicion of CCHFV infection in non-overt cases. Hematological testing is a routine procedure in clinical management and such prompt point-of-care indications could help in implementing quick interventions such as barrier nursing and patient isolation before outbreaks are confirmed or spread farther. Several recent studies support this proposal.^69^^–^^71^ Finally, the lack of extensive genetic variability between the Ugandan CCHFV strains is advantageous for application of a common vaccine as recently suggested.^72^

This study was, however, limited to hospitalized patients, perhaps representing the most severe cases. To fully understand the epidemiology of CCHFV in Uganda, more studies that include subclinical human infections, identification of other host animals, and tick vectors are needed.

## Supplemental tables


Supplemental materials
