# Supplementary material for: Clinical and Molecular Epidemiology of Crimean-Congo Hemorrhagic Fever in Humans in Uganda, 2013–2019
Source: Am J Trop Med Hyg. 2021 Oct 18;106(1):88–98. doi: 10.4269/ajtmh.21-0685 (PMC8733546; doi:10.4269/ajtmh.21-0685)
Supplement: Supplementary file 1 [file tpmd210685.SD1.pdf]

Supplementary Table 1: Overview of Putative Recombination Locations

Segment	Recombinant	Major Parent	Minor Parent	Identified Breakpoint Region	Recombinantion Tests RDP	GENECONV	BootScan	MaxChi	Chimaera	SiScan	3Seq	Total Corrected MC probability	Warnings:
M	201900896*	201706463	201900910	1-700 (690-906bp) and 5003-5' end	$9.131*10^{-50}$	$6.272*10^{-22}$	$2.736*10^{-120}$	$1.134*10^{-29}$	$4.191*10^{-28}$	$8.809*10^{-85}$	$1.302*10^{-132}$	$2.736*10^{-120}$	*No coverage at 153-403, 498-470, 554-581bp; 3' base is 5003bp.
M	201900888	201600162-3	201706462	3' to 315bp	-	$2.216*10^{-21}$	$1.105*10^{-20}$	$1.099*10^{-2}$	$5.619*10^{-3}$	-	$3.292*10^{-8}$	-	Possible misalignment artifact, recombination may be attributed to a process other than recombination. 44 bp insertion in this region appeats to be an assembly artifact, not seen in other CCHF genomes.
L	201900913	201900896	201900901	1-230bp	$2.620*10^{-2}$	$8.361*10^{-3}$	$5.784*10^{-4}$	-	-	$6.261*10^{-3}$	-	-	Recombination may be attributed to a process other than recombination. Only one parental sequence in triplet.
